# Metagenomic analysis evidences a core virome in *Anopheles darlingi* from three contrasting Colombian ecoregions

**DOI:** 10.1371/journal.pone.0320593

**Published:** 2025-04-30

**Authors:** Juan C. Hernandez-Valencia, Giovan F. Gómez, Margarita M. Correa

**Affiliations:** 1 Grupo de Microbiología Molecular, Escuela de Microbiología, Universidad de Antioquia, Medellín, Colombia; 2 Dirección Académica, Escuela de Pregrados, Universidad Nacional de Colombia, Sede de La Paz, La Paz, Colombia; National Institute of Agricultural Technology (INTA), ARGENTINA

## Abstract

*Anopheles darlingi* is a main malaria vector in the neotropical region, but its viral component is not well studied, especially in the neotropics. This work aimed to analyze the virome in *Anopheles darlingi* from malaria endemic regions of Colombia. Specimens were collected from the Bajo Cauca, Chocoan Pacific and northwestern Amazonas regions and analyzed using an RNA-Seq approach. Results revealed a variety of RNA viral sequences with homology to those of Insect-Specific Viruses belonging to *Rhabdoviridae*, *Partitiviridae*, *Metaviridae*, *Tymoviridae*, *Phasmaviridae*, *Totiviridae*, *Ortervirales* and *Riboviria*. Despite geographical and ecological differences among regions, the *An. darlingi* viral composition remains consistent in different areas, with a core group of viral operational taxonomic units-vOTUs shared by the populations. Furthermore, diversity analysis uncovered greater dissimilarities in viral sequence among mosquitoes from geographically distant regions, particularly evident between populations located at both sides of the Andes Mountain range. This study provides the first characterization of the metavirome in *An. darlingi* from Colombia and lays the foundation for future research on the complex interactions among viruses, hosts, and microbiota; it also opens a new line of investigation on the viruses in *Anopheles* populations of Colombia.

## Introduction

*Anopheles darlingi* is the most important malaria vector in the Neotropics, with a widespread geographical distribution covering southern Mexico to northern Argentina [1]. Traditionally, research on *An. darlingi* has focused on its role as a malaria vector and its interaction with the *Plasmodium* parasite [1,2]. Recently, its microbiota has been explored, but its viral component remains poorly researched [3–5].

The characterization of viruses within *Anopheles* genus contributes to identifying arboviruses and insect-specific viruses (ISVs) [6]. While traditional methods like isolation and molecular detection have been useful in achieving this goal, they have limitations, such as high costs and technical requirements [7]. In recent years, advances in sequencing technologies have enabled the study of viral communities in arthropods and contributed to uncovering the prevalence of ISVs with a host range restricted to insects [7,8]. The abundance and wide distribution of ISVs and their potential to interfere with arbovirus transmission have fueled a growing interest in evaluating the viral communities circulating in mosquitoes [9,10].

In Colombia, *An. darlingi* is found in different ecoregions and shows genetic differences between populations from the northwest and southeast, separated by the Andes mountains [11,12]. This species is one of the main malaria vectors in the country [1,12] and has a high degree of anthropophilic behavior [13] becoming a potential virus vector. However, no studies on the diversity and abundance of the metavirome in *Anopheles* mosquitoes in Colombia have been reported to date [6]. Given the existing gap in knowledge regarding the composition and diversity of viruses in neotropical *Anopheles*, this study employed a meta-transcriptomics approach to explore the composition and abundance of RNA virus sequences within *An. darlingi* in Colombia. For the first time, this study characterized the viral composition of *An. darlingi* from three different ecoregions at both sides of the Andes Mountain range in Colombia.

## Materials and methods

### Collection sites

**Fig 1:**
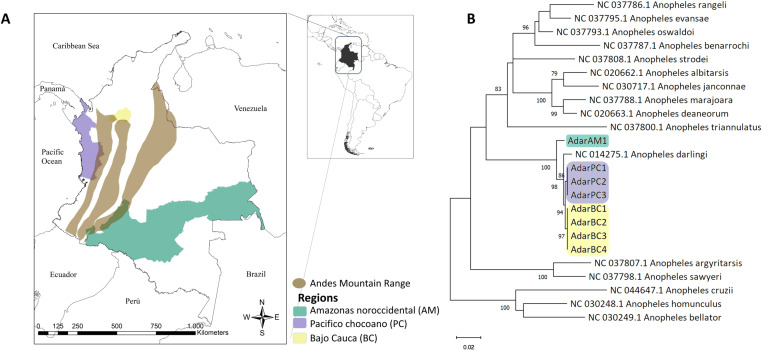
Sampling location and species confirmation. (A) Sampling regions for *Anopheles darlingi* natural populations in Colombia. (B) Confirmation of mosquito species based on a maximum likelihood phylogeny of the Cytochrome C Oxidase Subunit 1 (COX-1) sequences.

*Anopheles darlingi* mosquitoes were collected from three regions in Colombia: Bajo Cauca (BC) in the northwest, Chocoan Pacific (PC) in the west, and northwestern Amazonas (AM) in the south of the country. These regions are situated respectively in the Magdalena–Urabá, Chocó–Darién, and Negro–Branco humid forest ecoregions [14]. The PC and BC regions are situated west and northwest of the Andes mountains, respectively, while AM is located southeast of this mountain range (Fig 1A and S1 Table).

### Mosquito collection and processing

The collection of *Anopheles* mosquitoes followed the recommendations outlined in the WHO Training Manual on Malaria Entomology [15]. The permit for mosquito collection was granted by a Colombia national authority (Autoridad Nacional de Licencias Ambientales - ANLA), under the Framework Permit for the Collection of Wild Species Specimens for Non-Commercial Scientific Research (Resolution 0424, 2014). No specific permits were required to access the regions and localities to be sampled. Collectors read and signed a written informed consent approved by a Bioethics Committee of the Universidad de Antioquia (CBEIH-SIU, No. 18-35-810). Female mosquitoes were collected using protected human landing catches, while males were collected while resting on barrier screens (polyethylene shade cloth netting) in peridomicile, approximately 10 meters from the house. Subsequently, the collected mosquitoes were sacrificed with ethyl ethanoate and identified using a taxonomic key for *Anopheles* [16]. The mosquitoes were preserved in RNA-shield (Zymo Research), transported to the laboratory and stored at -80°C until further analysis.

To ensure data reproducibility and comparability, mosquito processing adhered to the recommendations of the Mosquito Microbiome Consortium [17]. Pools for RNA-Seq were formed with fifteen *An. darlingi* mosquitoes from the same locality; female mosquito pools were formed after visually assessing unfed and non-gravid specimens (S2 Table). Analyses of mosquito parity and age determination were omitted to maintain sample integrity. Molecular species assignation was confirmed using COX-1 sequences obtained from each pool, queried against an *Anopheles* genus COX-1 reference database obtained from NCBI repositories, using the BlastN tool v.2.3 (https://blast.ncbi.nlm.nih.gov/Blast.cgi) with an e-value < 10–5. Subsequently, a maximum likelihood-based phylogeny was constructed using reference *Anopheles* COX-1 sequences within the subgenera *Kerteszia* and *Nyssorhynchus*.

### RNA extraction, library preparation and sequencing

The pools were sent to a sequence facility for sample processing, library preparation and sequencing, as follows. Whole mosquitoes from each pool were homogenized in 750 μl of TRIzol (Thermo Fischer Scientific, Waltham, MA, USA) using tungsten carbide beads in a TissueLyser II (Qiagen). This homogenized product was then used for total RNA extraction, which was performed with QIAzol lysis reagent (Qiagen). The resulting RNA extracts were subsequently purified using RNeasy Mini Kit columns (Qiagen). Quantity and RNA integrity number (RIN >7) were determined using an Agilent 2100 Bioanalyzer (Agilent Technologies). Subsequently, the cDNA library was prepared with the Illumina TruSeq stranded kit for total RNA (Illumina). The Ribo-Zero-Gold kit (Illumina) was used to enrich non-ribosomal RNA. Sequencing was conducted in paired-end mode on a NovaSeq6000 (Illumina).

### Bioinformatic analysis

The quality of raw sequence data was assessed using FastQC v. 0.11.9 [18], and bases with low scores (Phred <20) were trimmed using Trimmomatic tool v. 0.38 [19]. To exclude sequenced reads derived from the host and discard potential endogenous viral elements, the quality-filtered reads were mapped to the *An. darlingi* reference genome idAnoDarlMG_H_01 (GCF_943734745.1) using Bowtie2 v. 2.5.0 with default parameters [20]. Reads not mapped to the genome were *de novo* assembled using MetaSPAdes v. 3.15.3 with *k-mers* 21, 33, 55 and 77 [21]. Finally, redundant sequences were removed by discarding contigs that shared 95% identity in more than 80% of the sequence using CD-HIT-est with default parameters [22], retaining the largest one for subsequent analyses.

### Viral sequences discovery and characterization

Read counts per viral taxon were obtained by querying quality-filtered reads against the NCBI BLAST nr+euk database using Kaiju tool [23]. For this analysis, Greedy mode was used with SEG filter, minimum match length = 11, minimum match score = 75, and allowed discrepancies = 5. The identification of viral sequences in each pool was carried out by querying non-redundant contigs (*nr-contigs*) against the protein version of the Reference Viral Database (RVDB) available at https://rvdb-prot.pasteur.fr/ [24]. For this query, DIAMOND tool v. 2.0.8 was employed with an e-value < 10^-5, and default parameters [25].

In addition, peptide sequences from open reading frames in *nr-contigs* were obtained using VirtualRibosome tool v. 2.0 [26]. Subsequently, the amino acid (aa) sequences were queried against different profiles of RNA-dependent RNA polymerase (RdRp) using Hidden Markov Models (HMM) available in the Pfam database (https://www.ebi.ac.uk/interpro) [27]: Birnaviridae-like: Birna_RdRp [PF04197.15], Bunyavirales-like: Bunya_RdRp [PF04196.15], Flaviviridae-like: Flavi_NS5 [PF00972.23], Narnaviridae-like: Mitovir_RNA_pol [PF05919.14], Mononega- and Chuviridae-like: Mononeg_RNA_pol [PF00946.22], Picornavirales-like and Nidovirales-like: RdRP_1 [PF00680.23], Tymovirales-like and Hepe-Virga-like: RdRP_2 [PF00978.24], Tombusviridae-like and Nodaviridae-like: RdRP_3 [PF00998.26], Toti-, Luteo-, and Sobemoviridae-like: RdRP_4 [PF02123.19], Reoviridae-like: RdRP_5 [PF 07925.14], Orthomyxoviridae-like: Flu_PB1 [PF00602], RVT_1 [PF00078.30], RVT_2 [PF07727.17], Arenaviridae-like: Arena_RNA_pol [PF06317]. Taxonomic assignment of viral sequences followed sequence-based taxonomy guidelines by the International Committee on Taxonomy of Viruses (ICTV) [28,29].

### Viral sequence annotation and phylogenetic analysis

For functional annotation of viral sequences, the amino acid sequence of identified ORFs were queried against the NCBI Conserved Domains Database (CDD) using RPS–BLAST (e-value < 10^-3, other parameters were kept at default) [30]. When no results were obtained with CDD, the sequences were queried against the Protein Data Bank (PDB) database using HHpred (e-value < 10^-3, other parameters were kept at default) [31].

The phylogeny of viral sequences was inferred using maximum likelihood based on RdRp or capsid amino acid sequences [32]. A phylogenetic tree was constructed by querying related viral sequences in the NCBI database. Multiple sequence alignments were performed using MUSCLE [33], and the alignments were manually checked to avoid misalignments or ambiguities. The best-fit substitution model for each alignment was selected using the Bayesian Information Criterion (BIC). Phylogenetic trees and optimal substitution models were generated in MEGA v.11 [34], with statistical support based on 500 bootstrap replicates.

### Viral sequence abundances and diversity

Metavirome composition among different *An. darlingi* populations was evaluated using two abundance matrices: 1) a read-based matrix, constructed from reads identified as viral and taxonomically assigned, using the Kaiju tool, and 2) a contigs-based matrix, built from mapping reads onto identified viral non-redundant contigs (henceforward referred to as viral OTUs or vOTUs) using BBmap tool [35] with a minimum threshold of 95% nucleotide identity. The read-based matrix was normalized by considering the size of each sequenced library and the vOTUs-based matrix was normalized by the length of the vOTUs and the size of each library. To avoid false positives, vOTUs in which reads were not mapped in more than 75% of the sequence were assigned a zero value in the abundance matrix [36].

Diversity indices were determined from both abundance matrices. Alpha diversity was assessed using the Shannon-Weaver (H) and Richness (S) indices. Beta diversity among the metaviromes of *An. darlingi* populations was calculated using Bray-Curtis (BC) dissimilarity index and Euclidean distances. The statistical significance between regions was assessed using an analysis of similarities (ANOSIM) and permutational multivariate analysis of variance (PERMANOVA). To identify the viral taxa and vOTUs contributing the most to the dissimilarity between groups, similarity percentage analysis (SIMPER) was applied. Alpha and beta diversity indices and statistical tests were performed and visualized using the metagenome-Seq [37], Vegan [38], and ggplot2 [39] packages in R [40]; SIMPER analysis was determined using PAST v. 4 [41].

## Results

In total, nine pools of non-gravid and unfed females were processed, except for the only pool of males collected during fieldwork (AdarPC3). In each pool, *An. darlingi* COX-1 sequences were identified (>99% nucleotide sequence identity) (Fig 1B). Each pool generated an average of 39,683,990 quality-filtered reads, ranging from 34,103,154–44,581,293. Around 78% to 87% of reads mapping to the *An. darlingi* genome were discarded (S1 Fig). The background layer used in this figure was obtained from the web site Humanitarian Data Exchange (HDX), link: https://data.humdata.org/dataset/geoboundaries-admin-boundaries-for-colombia.

### Overview of the mosquito viral sequences

**Fig 2:**
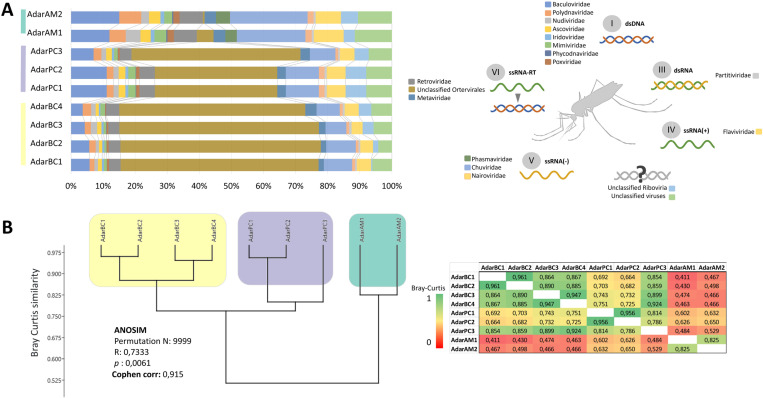
Viral classification and Bray-Curtis distances of quality-filtered reads in *Anopheles darlingi* natural populations from Colombia. (A) Classification of quality-filtered reads by viral taxon. (B) Dendrogram and distance matrix of the Bray-Curtis dissimilarity between samples grouped by the study region. AdarAM: northwestern Amazonas, AdarPC: Chocoan Pacific, AdarBC: Bajo Cauca.

The taxonomically classified viral reads were distributed among 15 families and three groups not classified at the viral family level. The highest proportion of reads was assigned to the group of unclassified *Ortervirales* (48.9%), followed by the families *Chuviridae* (10%), *Baculoviridae* (7.3%), and a group of “unclassified viruses” (6.9%) (Fig 2A). The *de novo* assembly yielded between 17000 and 19000 contigs per pool, with 24 vOTUs identified across the pools. All sequences exhibited identity with previously detected insect viral sequences and belonged to the families *Rhabdoviridae*, *Partitiviridae*, *Metaviridae*, *Tymoviridae*, *Phasmaviridae* and *Totiviridae*, and *Ortervirales* and *Riboviria* groups (Table 1). S3 Table displays the protein domains detected in identified viral sequences.

**Table 1:** Viral sequences detected in *Anopheles darlingi* natural populations from Colombia.

Closest related sequence (BlastX)	Pool	nr-*contig* length (nt)	aa Id (%)	Sequence type	Reported host/ Country	Virus taxon	Name given in this job^*^
Culex tritaeniorhynchus rhabdovirus (BBQ05110.1)	AdarBC1	192	46.2	Spike glycoprotein	*Culex tritaeniorhynchus*/ Japan	*Rhabdoviridae*	Anopheles darlingi rhabdoviridae-like sequence 1
Culex tritaeniorhynchus rhabdovirus (BAU46576.1)	AdarBC3	515	67.2	RdRp	*Culex tritaeniorhynchus*/ Japan	*Rhabdoviridae*	Anopheles darlingi rhabdoviridae-like sequence 2
Culex tritaeniorhynchus rhabdovirus (QTW97817.1)	AdarPC3	655	65.2	RdRp	*Culex tritaeniorhynchus*/ China	*Rhabdoviridae*	Anopheles darlingi rhabdoviridae-like sequence 3
Cimo rhabdovirus I (MZ202301)	AdarBC1	519	65.2	RdRp	*Culex* sp./ Costa de Marfil	*Rhabdoviridae*	Anopheles darlingi rhabdoviridae-like sequences 4
Atrato Partiti-like virus 2 (QHA33903.1)	AdarBC1, AdarBC2, AdarBC3, AdarBC4, AdarPC1, AdarPC2, AdarPC3, AdarAM1, AdarAM2	1009	62.8	Capsid	*Anopheles darlingi*/ Colombia	*Partitiviridae*	Anopheles darlingi_partitivirus-like sequence 1
Atrato Partiti-like virus 2 (QHA33903.1)	AdarBC1, AdarBC1	1.160	92.2	Capsid	*Anopheles darlingi*/ Colombia	*Partitiviridae*	N/A
Atrato Partiti-like virus 2 (QHA33902.1)	AdarBC1, AdarBC2	1.820	94.2	RdRp	*Anopheles darlingi*/ Colombia	*Partitiviridae*	N/A
Chibugado virus (QHA33695.1)	AdarBC1, AdarBC3, AdarBC4, AdarPC1, AdarPC2, AdarPC3, AdarAM1	5.432	57.9	Polyprotein	*Psorophora albipes*/ Colombia	*Metaviridae*	N/A
Atrato Retro-like virus (QHA33696.1)	AdarBC1, AdarPC2, AdarAM1	5.472	99.8	Polyprotein	*Anopheles darlingi*/ Colombia	unclassified Ortervirales	N/A
Atrato Retro-like virus (QHA33696.1)	AdarPC3	6.375	77.4	Polyprotein	*Anopheles darlingi*/ Colombia	unclassified *Ortervirales*	Anopheles darlingi Retro-like virus 1
Atrato Retro-like virus (QHA33696.1)	AdarBC2, AdarBC4	1.474	60.5	Polyprotein	*Psorophora albipes*/ Colombia	unclassified *Ortervirales*	Anopheles darlingi Retro-like virus 2
Hubei odonate virus 15 (APG79146.1)	AdarBC1, AdarBC2, AdarBC3, AdarBC4, AdarPC1, AdarPC2, AdarPC3, AdarAM1, AdarAM2	3.608	30.3	hypothetical protein	Odonata/ China	unclassified *Riboviria*	Anopheles darlingi orbivirus-like sequence
Wuhan insect virus 23 (YP_009329883.1)	AdarBC1, AdarPC3	501	63.3	hypothetical protein	insects/ China	unclassified *Riboviria*	Anopheles darlingi virus-derived sequence
Aedes aegypti To virus 1 (QPF16707.1)	AdarBC2, AdarBC3, AdarBC4, AdarPC1, AdarPC2, AdarPC3, AdarAM2	5.264	67.1	RdRp	*Aedes aegypti*/ Brazil	unclassified *Riboviria*	N/A
Aedes aegypti To virus 2 (QPF16710.1)	AdarBC1, AdarBC2	4.562	47.9	RdRp	*Aedes aegypti*/ Brazil	unclassified *Riboviria*	Anopheles darlingi To virus 1
Aedes aegypti To virus 2 (QPF16710.1)	AdarBC3, AdarBC4, AdarPC1, AdarPC2, AdarPC3, AdarAM1, AdarAM2	4.241	44.7	RdRp	*Aedes aegypti*/ Brazil	unclassified *Riboviria*	Anopheles darlingi To virus 2
Chaq virus-like 1 (AOR51384.1)	AdarBC1, AdarBC2, AdarBC3, AdarBC4, AdarPC1, AdarPC2, AdarPC3, AdarAM2	3.374	62.5	ORF1	*Anopheles gambiae*/ Liberia	unclassified viruses	Anopheles darlingi Chaq virus-like
Murindo virus (QHA33700.1)	AdarPC2	3.801	97.3	Polyprotein	*Anopheles darlingi*/ Colombia	*Tymoviridae*	N/A
Kaiowa Virus (ASV45863.1)	AdarBC3	1.548	65.7	Glycoprotein	*Stegomyia albopicta*/ Brazil	unclassified viruses	Anopheles darlingi virus-derived sequence 3
Anopheles triannulatus orthophasmavirus RNA3 (YP_010086187.1)	AdarAM2	2.124	90.4	Nucleocapsid, ORF3	*Anopheles triannulatus*/ Brazil	*Phasmaviridae*	Anopheles darlingi orthophasmavirus like sequence
Anopheles triannulatus orthophasmavirus RNA3 (YP_010086187.1	AdarAM1, AdarAM2	1.830	91.3	Nucleocapsid, ORF3	*Anopheles triannulatus*/ Brazil	*Phasmaviridae*	N/A
Anopheles triannulatus orthophasmavirus RNA2 (YP_010086190.1)	AdarAM1, AdarAM2	1.346	88.4	Glycoprotein	*Anopheles triannulatus*/ Brazil	*Phasmaviridae*	N/A
Anopheles triannulatus orthophasmavirus RNA1 (YP_010086189.1)	AdarAM1	4.452	95.6	RdRp	*Anopheles triannulatus*/ Brazil	*Phasmaviridae*	N/A
Murri virus (QHA33713.1)	AdarAM1, AdarAM2	1.577	30.6	Capsid	*Coquillettidia venezuelensis*/ Colombia	*Totiviridae*	Anopheles darlingi totiviridae-like sequence

* A name was provided for virus sequences that met the sequence-based taxonomy guidelines for new viruses established by ICTV. N/A: sequences that retained previously assigned names.

### Double-stranded RNA viruses

**Fig 3:**
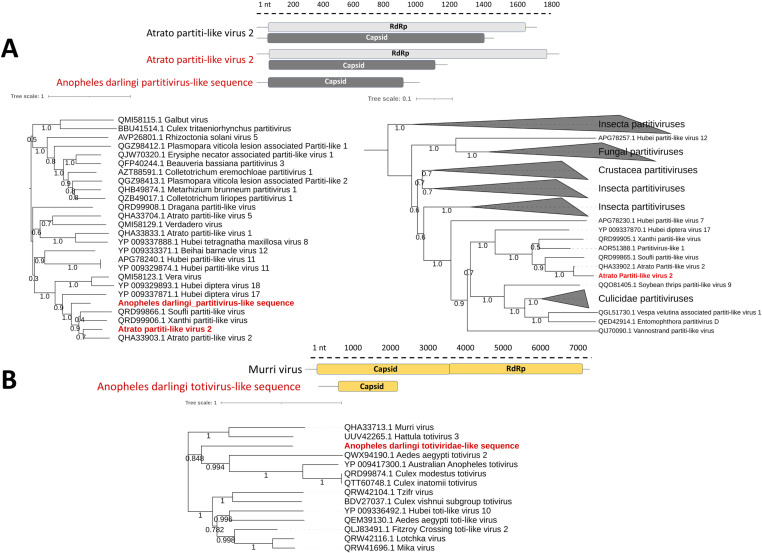
Genome organization and maximum likelihood phylogenies of dsRNA viruses. (A) Maximum likelihood phylogeny of *Partitiviridae* sequences based on RNA-dependent RNA polymerase (RdRp) (right) and capsid protein (left) sequences. The phylogenetic trees were constructed using the Le-Gascuel (LG+G+I) model. (B) Maximum likelihood phylogeny of *Totiviridae* sequences based on capsid protein sequences. The tree was constructed using the Le-Gascuel (LG+G) model. Virus sequences identified in this study are indicated in red text.

The query against RVDB and conserved domains database resulted in the detection of four vOTUs related to viruses with double-stranded RNA genomes (dsRNA). Among these vOTUs, three were associated with viruses of the *Partitiviridae* family; one vOTU exhibited 94% aa identity to the RdRp sequence of the Atrato Partiti-like virus 2 (GenBank accession: QHA33902), while the other two showed 92% and 62% aa identities to the capsid sequence of this virus. The remaining vOTU displayed 33% aa identity to the capsid sequence of the Murri virus (GenBank accession: QHA33902), belonging to the family *Totiviridae* (Table 1 and S3 Table). Phylogenetic analysis of the aa sequences encoding RdRp and capsid revealed their relationship with other viruses from the families *Partitiviridae* and *Totiviridae*, previously identified in various *Anopheles*, *Aedes*, *Ochlerotatus* and *Culex* species from America, Africa and southeastern Europe [42] (Fig 3).

Based on criteria for sequence-based classification of the *Partitiviridae* [43] and *Totiviridae* families [44], this study recovered genome sequence of the Atrato Partiti-like virus 2, a virus of the order *Durnavirales* previously detected in *An. darlingi* on the banks of the Atrato River in Colombia; and also, two partial sequences encoding the capsid protein of a partitivirus and a totivirus, named in this work as Anopheles darlingi partitivirus-like sequence and Anopheles darlingi totiviridae-like sequence, respectively (Table 1, Fig 3).

### Negative-sense, single-stranded RNA viruses

**Fig 4:**
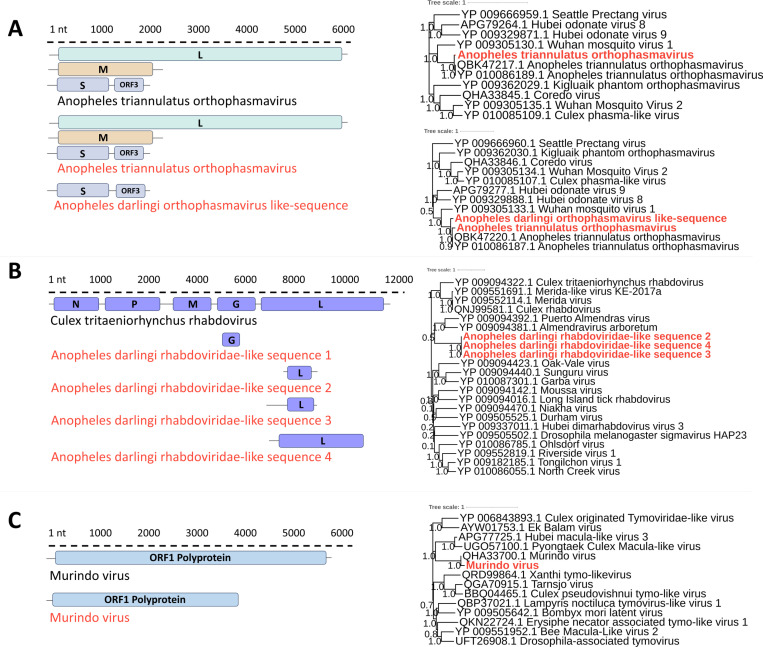
Genome organization and maximum likelihood phylogenies of ssRNA- and ssRNA+ viruses. (A) Maximum likelihood phylogeny of *Phasmaviridae* sequences based on RNA-dependent RNA polymerase (RdRp) (top) and nucleocapsid (bottom) protein sequences. Phylogenetic trees were constructed using the Le-Gascuel models (LG+G+F for RdRp and LG+G+I for nucleocapsid). (B) Maximum likelihood phylogeny of *Rhabdoviridae* sequences based on RdRp protein sequences, constructed using the Whelan and Goldman (WAG+G) model. (C) Maximum likelihood phylogeny of *Tymoviridae* sequences based on polyprotein sequences, constructed using the Le-Gascuel (LG+G+F) model. Virus sequences identified in this study are highlighted in red text.

Eight vOTUs related to viruses with a negative single-stranded RNA genome (ssRNA-) were detected. Four vOTUs presented identity against viral sequences of the *Phasmaviridae* family; two showed an identity of 90% and 91% (aa) with the segment encoding the nucleocapsid of Anopheles triannulatus orthophasmavirus (NCBI Reference Sequence: YP_010086187), a third showed a 88% identity with the glycoprotein aa sequence of this virus (NCBI Reference Sequence: YP_010086190), and a fourth showed 95.6% (aa) identity compared to a segment encoding RdRp (NCBI Reference Sequence: YP_010086189). The phylogeny based on amino acid sequences of RdRp and the nucleocapsid clustered with sequences of the genus *Orthophasmavirus* recently identified from insects, most of them culicids [45–49] (Fig 4A). Following the criteria for species demarcation within the *Phasmaviridae* family [50], these results indicate recovery of three segments of the genome of Anopheles triannulatus orthophasmavirus, family *Phasmaviridae*, order *Bunyavirales*. Additionally, a sequence corresponding to the M segment of an orthophasmavirus was identified, named here as Anopheles darlingi orthophasmavirus-like sequence.

Moreover, four vOTUs associated with the *Rhabdoviridae* family were detected. One exhibited a 35% aa identity with a segment of the G protein sequence of the Culex tritaeniorhynchus rhabdovirus, while two displayed aa sequence identities of 48% and 57% against sequences encoding the L protein of this virus. Additionally, a fourth vOTU showed a 65% aa identity with the L protein of Cimo rhabdovirus I. These viruses, Culex tritaeniorhynchus rhabdovirus and Cimo rhabdovirus I, have been previously identified in natural populations of *Culex* mosquitoes in Asia and Africa [46,51]. The genome fragments of these four viral sequences are designated here as Anopheles darlingi rhabdoviridae-like sequences 1–4 (Fig 4B).

### Positive-sense, single-stranded RNA viruses

In the dataset obtained from the Chocoan Pacific *An. darlingi* natural population, a vOTU was identified, showing 97% aa identity with the Murindo virus polyprotein (GenBank accession: QHA33700) (Table 1 and S3 Table). A phylogenetic analysis of the polyprotein amino acid sequence revealed a distinct clade containing the Murindo virus sequence and sequences from Culex Macula-like virus and Hubei macula-like virus 3. These two tymoviruses have been previously detected in the order Diptera in South Korea and Araneae in China [52,53]. Phylogenetically, these viruses are closely related to other members of the *Tymoviridae* family detected in insects, excluding Erysiphe necátor-associated tymo-like virus 1, which was identified in a fungus (Fig 4C). Following the demarcation criteria for species in the family *Tymoviridae* [44], the sequence identified in this study corresponds to a partial genome of the Murindo virus, *Tymoviridae* family, *Orthornavirae* order. This virus was recently detected in *An. darlingi* in Colombia (GenBank accession: MN661046).

### Positive-sense, single-stranded RNA-RT viruses

**Fig 5:**
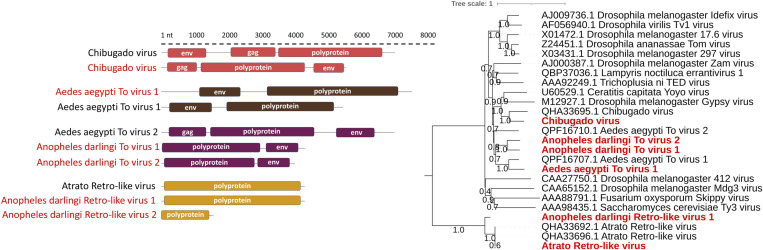
Genome organization and maximum likelihood phylogenies of positive-sense, single-stranded RNA-RT viruses detected in this study. Maximum likelihood phylogeny of *Ortervirales* order sequences based on polyprotein amino acid sequences. The phylogenetic tree was constructed using the general reverse transcriptase model (rtREV+G+I+F). Virus sequences identified in this study are shown in red text.

In this study, seven vOTUs were identified with sequence identity similar to those of the *Ortervirales* order. Two of these vOTUs displayed aa identities of 44% and 48% with the polyprotein of Aedes aegypti To virus 2 (GenBank accession: QPF16710). Another vOTU demonstrated 67% identity with the Aedes aegypti To virus 1 polyprotein (GenBank accession: QPF16707). A fourth vOTU showed a 58% aa identity with the Chibugado virus polyprotein (GenBank accession: QHA33694) (Table 1 and S3 Table). Phylogenetic analysis of Gag-Pol polyprotein sequences revealed clustering with other viruses of the *Errantivirus* genus, primarily identified in insects, especially Diptera (Fig 5). Although species demarcation criteria within the *Metaviridae* family are not well-defined, ICTV suggests that species in this family should share less than 50% Gag polyprotein sequence identity [54]. Following this criterion and considering phylogeny results, sequences corresponding to Aedes aegypti To virus 1 and Chibugado virus were identified, along with two new viruses named here Anopheles darlingi To virus 1 and Anopheles darlingi To virus 2. Furhtermore, three vOTUs showed 99%, 77% and 60% aa identities against the Atrato Retro-like virus polyprotein (Table 1). Phylogenetic analysis of the Gag polyprotein grouped these vOTUs with the Atrato Retro-like virus, forming a separate clade from the one of *Metaviridae* family viruses (Fig 5). From these three viral genome sequences recovered of the *Ortervirales* order, two are new and are named here, Anopheles darlingi Retro-like virus 1 and Anopheles darlingi Retro-like virus 2.

### Viral sequence abundances and diversity

**Fig 6:**
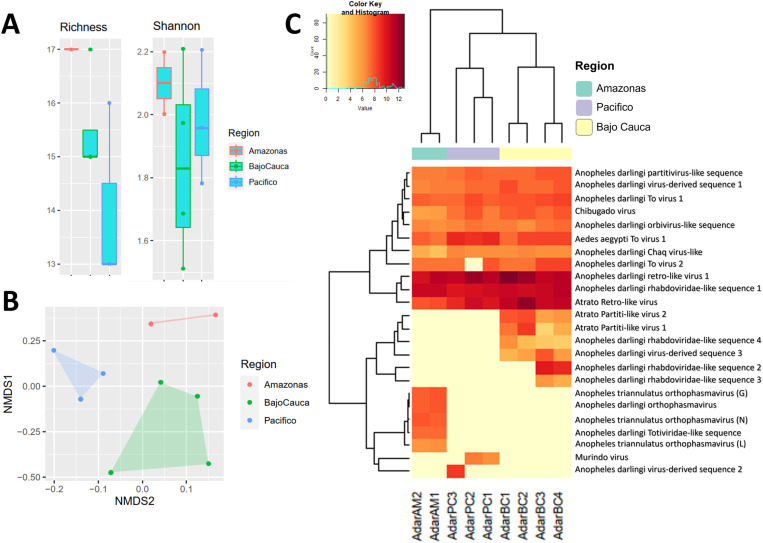
Alpha and beta diversity of vOTUs detected in *An. darlingi* natural populations from Colombia. (A) Shannon index and Richness of vOTUs across study regions (Kruskal‒Wallis test ****p**** > 0.05). (B) Non-metric Multi-dimensional Scaling based in Bray-Curtis distances across study regions (Stress= 0.066, PERMANOVA test ****p**** < 0.009, R2 = 3.56). (C) Heatmap illustrating normalized read counts and Euclidean-based clustering across study regions (ANOSIM test, **p** < 0.05, **R** = 0.61). The heatmap shows vOTU abundance, with dark colors indicating higher abundance and light colors representing lower abundance.

The Shannon, Richness and Evenness indices revealed no significant differences between regions (AM, PC and BC) (Kruskal‒Wallis tests *p* > 0.05). This indicates uniform distribution of alpha viral diversities within *An. darlingi* populations, irrespective of the geographic location (Fig 6A). Conversely, the beta diversity analysis, based on reads per viral taxon and vOTUs, revealed a higher similarity in the metavirome structure among *An. darlingi* populations within the same region compared to populations among regions (Fig 6B and 6C). Notably, a higher similarity was observed between the Chocoan Pacific and Bajo Cauca than between Bajo Cauca and northwestern Amazonas, as well as between Chocoan Pacific and northwestern Amazonas (Figs 2B and 6C). Interestingly, the only male pool (AdarPC3), from the Chocoan Pacific region exhibited higher similarity with *An. darlingi* female pools from the same region. ANOSIM and PERMANOVA tests indicated statistically significant differences between groups (PC, BC and AM). Similarity percentage analysis showed that the taxon “Unclassified *Ortervirales*” and vOTUs Anopheles darlingi retro-like virus 1, Atrato retro-like virus and Anopheles darlingi rhabdoviridae-like sequence 1 were the main contributors to differences observed among populations.

Finally, this study found that different regions share a common set of vOTUs, even though with variable abundances (Fig 6C). These include Anopheles darlingi_partitivirus-like sequence related to the *Partitiviridae* family, Anopheles darlingi retro-like virus 1, Anopheles darlingi To virus 1, Aedes aegypti To virus 1, Chibugado virus, Atrato retro-like virus and Anopheles darlingi retro-like virus 2, with the latter two showing high abundance. Moreover, Anopheles darlingi–virus-derived sequence 1, Anopheles darlingi orbivirus-like sequence, Anopheles darlingi Chaq virus-like and Anopheles darlingi rhabdoviridae-like sequence 1 were common across regions, with the latter exhibiting a high abundance in all populations.

## Discussion

This study represents the first characterization of the metavirome structure in *Anopheles* mosquitoes from Colombia; specifically, natural populations of *An. darlingi*, a malaria vector of significant epidemiological importance. The analysis revealed 24 vOTUs, all exhibiting amino acid sequence identities with viruses previously detected in insects, most of them culicids. Phylogeny confirmed their close relationship to putative insect-specific viruses of culicids [6,55].

Among the *Partitiviridae* family, two vOTUs, Atrato Partiti-like virus 2 and Anopheles darlingi_Partitivirus-like sequence, were assembled. Atrato Partiti-like virus 2 was previously identified in *An. darlingi* in Colombia (unpublished data), and viruses within the same phylogenetic clade have been found in *Anopheles gambiae* in Liberia [42] and in *Culiseta longiareolata* and *Coquillettidia richiardii* in Greece [56]. According to ICTV, the *Partitiviridae* family has a host range limited to plants, fungi and protozoa [43]; however, in recent years, partitviruses have been recurrently identified in insects [53], including culicids, such as *Anopheles* mosquitoes [8,57]. Furthermore, this study recovered the partial genome of Murindo virus, belonging to the *Tymoviridae* family, initially identified in *An. darlingi* from Colombia (GenBank accession: QHA33700). The sequences of Hubei macula-like virus 3 and Pyongtaek Culex Macula-like virus clustered in the same phylogenetic clade as the Murindo virus; these viral sequences have been detected in *Anopheles* and *Culex* species from Oceania and Asia [52,53]. The detection of the Murindo virus in two pools from the Chocoan Pacific region, along with its previous identification in *An. darlingi* in Colombia, suggests active transmission or a close ecological relationship with this virus [58]. Though, its acquisition through ingestion of plant sugars is another possible route of transmission to the mosquito [59]. Although *Tymoviridae* is recognized by ICTV as a family of plant viruses, viral replication has been demonstrated in cicadas, suggesting their potential role as vectors [60]. Furthermore, a tymoviridae-like virus was recently isolated from *Culex* mosquitoes in Colombia [61].

This study also broadens the spectrum of host species for the *Phasmaviridae* family; for example, the vOTUs Anopheles triannulatus orthophasmavirus and Anopheles darlingi orthophasmavirus-like sequence were identified. Anopheles triannulatus orthophasmavirus has been found previously in *Anopheles triannulatus* and *Anopheles* sp. natural populations in the Brazilian Amazon region [45,62]. This virus was detected in localities within the Amazonian region, indicating its widespread distribution in *Anopheles* species of this region. Moreover, Anopheles triannulatus orthophasmavirus clustered in the same phylogenetic clade as Wuhan mosquito virus 1, identified in *Anopheles sinensis* in China [49], and *Anopheles* spp. in Cambodia and Senegal [63]; this suggests a wide distribution of this viral family within the *Anopheles* genus [8].

Three previously reported vOTUs in Culicidae from the Neotropics were among the seven Ortervirales identified. Within the *Metaviridae* family, Aedes aegypti To virus 1 and the Chibugado virus were identified; these viruses were previously detected in *Aedes aegypti* from Brazil [64] and *Psorophora albipes* from Colombia (GenBank accession: MN661043.1), respectively. The presence of *env* gene in the assembled genomes allowed their classification within the *Errantivirus* genus [54]. Noteworthy, the sequences identified in this study are phylogenetically related to errantiviruses previously detected in insects. One of these is closely related to the Drosophila melanogaster Gypsy virus, for which infective viral particle formation and horizontal transmission has been demonstrated [54]. Additionally, the Atrato Retro-like virus, in the *Ortervirales,* was detected; it was previously reported in *An. darlingi* and *Psorophora albipes* in Colombia (GenBank accession: MN661044 and MN661042). Of notice, Anopheles darlingi To virus 1, Anopheles darlingi To virus 2, Anopheles darlingi To virus 3, and Anopheles darlingi Gypsy virus are the first viral retroelements of the *Metaviridae* family reported in *An. darlingi*; deserving further research to clarify their roles and potential for horizontal transmission [54].

Finally, four vOTUs were classified in the *Rhabdoviridae* family. These sequences exhibited amino acid identity with Culex tritaeniorhynchus rhabdovirus and Cimo rhabdovirus I, both previously identified in natural populations of *Culex* and *Anopheles* mosquitoes in Asia and Africa [46,51]. The *Rhabdoviridae* family is noteworthy for its extensive global distribution, particularly among the *Aedes*, *Culex* and *Anopheles* genera [6,8].

Related to the *An. darlingi* metavirome variation according to geographic regions, previous studies have shown the presence of a species-specific virome in mosquitoes, where putative ISVs are consistently identified across different populations [65–68], even spanning continents [66,69,70]. This group of viruses is known as the core virome, although there is currently active debate surrounding this concept [71,72]. In this study, 40% of identified vOTUs were shared among *An. darlingi* populations from different subregions. Furthermore, some vOTUs were previously detected in an *An. darlingi* population along the Atrato River in Colombia (viral sequences published on NCBI repository); specifically, the Atrato Retro-like virus was present in all populations from the study regions, the Atrato Partiti-like virus 2 was detected in the Bajo Cauca region, and the Murindo virus was identified in the Chocoan Pacific region. These findings suggest that a portion of the *An. darlingi* metavirome is common to various populations covering a broad geographic range in Colombia, supporting the hypothesis of a stable species or population-specific virome for culicid species across various geographic scales [65–67,71,72].

Although some viral sequences were shared among the *An. darlingi* natural populations, beta diversity analyses revealed statistically significant differences in metavirome structure. Read counts for each viral taxon and vOTU showed greater similarity within regions than between them. Percentage similarity analysis identified the Unclassified Ortervirales taxon and vOTUs of Anopheles darlingi Retro-like viru 1 and Atrato Retro-like virus as the principal contributors to dissimilarities among study populations. Except for the Amazonian populations, a greater proportion of reads were assigned to “Unclassified *Ortervirales*” group. Analyses also showed that the highest read counts were for vOTUs Atrato-Retro-like virus and Anopheles darlingi Retro-like virus 1; both belong to the *Ortervirales* order and were identified in all *An. darlingi* populations. Taxa with higher abundances often exhibit greater variances, contributing more significantly to group dissimilarities [73]. Consequently, the abundance of the *Ortervirales* component in its metavirome is responsible for the observed differences in viral sequence structure among Colombian *An. darlingi* natural populations. This virus order was also dominant on other insects species like *Aedes*, *Culex* and Chrysomelidae [64,74,75].

Amazonian *An. darlingi* populations exhibited lower viral sequence structure similarity than Bajo Cauca and Chocoan Pacific populations. Interestingly, the similarity between the latter two was higher. They are located in the Magdalena–Urabá and Chocó–Darién humid forest ecoregions, respectively [14], and are separated from the northwestern Amazonas (Negro–Branco humid forest ecoregion) by the Andean mountain range, which rises to an elevation of approximately 6000 meters above sea level [76]. Furthermore, the Bajo Cauca and Chocoan Pacific regions are divided by the Colombian western range, which extends towards the Colombian Caribbean coast in the Abibe mountain range, with elevations ranging from approximately 500–1000 meters above sea level [14,76]. Differences in viral community structure among *An. darlingi* populations and regions could be attributed to variation in ecological conditions, the existence of natural barriers separating populations and/or mosquito-local hosts interactions [77]. Accordingly, a recent study on *Culex*, *Aedes*, *Anopheles* and *Armigeres* mosquitoes revealed that environmental factors affect intraspecies virome variation. Additionally, mosquito interaction with nearby hosts significantly influences virome composition [78].

Regarding the single male pool obtained from the Chocoan Pacific region, its virus taxa and vOTUs composition were similar to the female pools from the same region. This finding agrees with observations from other studies where a similar metavirome composition was found in males and females of the same species and area [47,79]. Although understanding viral sequence structure differences based on mosquito sex would require larger sample sizes [80], collecting male *Anopheles* mosquitoes presents additional challenges and requires distinct sampling strategies [81].

Finally, a key challenge in virome studies, including ours, is distinguishing between actively replicating viruses and endogenous viral elements [82]. While the use of the high-quality *An. darlingi* genome (assembly “idAnoDarlMG_H_01”) significantly enhanced our ability to filter out endogenous viral sequences, it remains difficult to fully exclude them due to their genomic similarity to active viruses [82]. This limitation, combined with the small sample size, reduces the generalizability of our findings, particularly when assessing the influence of geographic location on viral diversity and richness. The logistical challenges of collecting *Anopheles* mosquitoes in Colombia further constrained our ability to gather more specimens. Future studies with more extensive sample sizes will be essential for a deeper understanding of the geographic and ecological factors shaping viral diversity in these mosquito populations. Nonetheless, despite these limitations, our study makes a valuable contribution to the understanding of the metavirome of *An. darlingi* in Colombia, offering important baseline data on the viral landscape of this key malaria vector.

## Conclusion

This study offers valuable insights into the virome of the Colombian *An. darlingi* populations. Results suggest that mosquitoes from closer geographical regions have similar viral-sequences composition and abundance. Conversely, populations located at further geographical distances showed higher differences in their viral diversity. Despite the detected viral diversity, a set of virus sequences were common among *An. darlingi* populations in Colombia. This study provides the first characterization of the metavirome in *Anopheles* from Colombia and establishes a foundation for exploring the complex interactions among viruses, hosts, and microbiota; it initiates a new line of research focused on viruses in *Anopheles* mosquitoes of Colombia. Furthermore, viral isolation and deep characterization will help to confirm their occurrence, understand their transmission dynamics and determine their potential pathogenic role in nature.

## Supporting information

S1 Fig Classification of quality-filtered reads. (A) Reads mapped onto the Anopheles darlingi reference genome; (B) Unmapped reads classified as microbial.
(TIF)

S1 Table Metadata from the localities where natural population of *Anopheles darlingi* were sampled. (PDF)

S2 Table Pools of *Anopheles darlingi* mosquitoes processed for sequencing. (PDF)

S3 Table Open reading frames and protein domains in the virus sequences detected in *An. darlingi* natural populations from Colombia. (PDF)
