# Supplementary figures and images for: Metagenomic analysis evidences a core virome in Anopheles darlingi from three contrasting Colombian ecoregions
Source: PLoS One. 2025 Apr 30;20(4):e0320593. doi: 10.1371/journal.pone.0320593 (PMC12043238; doi:10.1371/journal.pone.0320593)

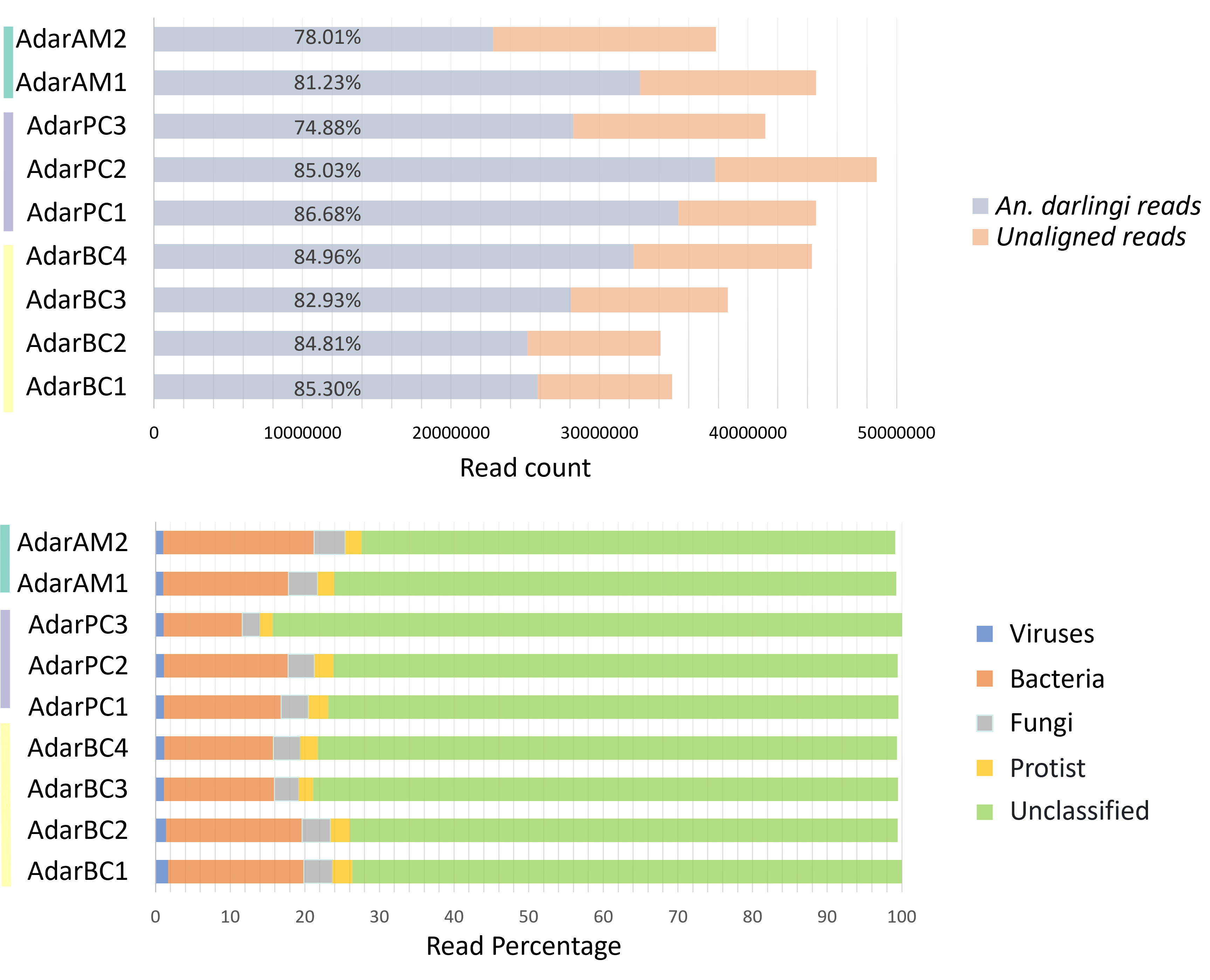

Supplement: S1 Fig — (A) Reads mapped onto the Anopheles darlingi reference genome; (B) Unmapped reads classified as microbial. (TIF) [file pone.0320593.s001.tif]
