# Supplementary material for: Metagenomic analysis evidences a core virome in Anopheles darlingi from three contrasting Colombian ecoregions
Source: PLoS One. 2025 Apr 30;20(4):e0320593. doi: 10.1371/journal.pone.0320593 (PMC12043238; doi:10.1371/journal.pone.0320593)
Supplement: S1 Table — (PDF) [file pone.0320593.s002.pdf]

S1 Table. Metadata from the localities where natural population of *Anopheles darlingi* was sampled.

Region/ Locality	Coordinates	T(°C)	RH (%)	m.a.s.l	Predominant landcovers
Bajo Cauca/ La Capilla	7°31'25,4"N 74°43'27,9"W	29,4	82	176	forest, water body, grass, bare soil,
Bajo Cauca/ Villa Grande	7°33'0,1"N 74°41'16,6"W	26,9	87	143	forest, water body, grass, bare soil
Pacífico / San Antonio	5°7' 49,4" N 76°41'25,2"W	31,1	78	85	forest, water body, shrub
Amazonas/ Caño Negro	2°45'59.6 N 72°04 22.7"W	26,9	86	171	grass, bare soil, forest
Amazonas/ Charras	2°47'11.3"N 71°56'56.3"W	24,4	89	165	grass, bare soil, forest

T: temperature, **RH**: relative humidity, **m.a.s.l**: meters above sea level.
