# Supplementary material for: Metagenomic analysis evidences a core virome in Anopheles darlingi from three contrasting Colombian ecoregions
Source: PLoS One. 2025 Apr 30;20(4):e0320593. doi: 10.1371/journal.pone.0320593 (PMC12043238; doi:10.1371/journal.pone.0320593)
Supplement: S2 Table — (PDF) [file pone.0320593.s003.pdf]

S2 Table. Pools of *Anopheles darlingi* mosquitoes processed for sequencing.

Region/ locality	Sex (n)	gonotrophic state / feeding status	pool ID
Bajo Cauca/ La Capilla	Female (15)	Not gravid / not fed	AdarBC1
Bajo Cauca/ La Capilla	Female (15)	Not gravid/ not fed	AdarBC2
Bajo Cauca/ Villa Grande	Female (15)	Not gravid/ not fed	AdarBC3
Bajo Cauca/ Villa Grande	Female (15)	Not gravid/ not fed	AdarBC4
Pacífico/ San Antonio	Female (15)	Not gravid/ not fed	AdarPC1
Pacífico/ San Antonio	Female (15)	Not gravid/ not fed	AdarPC2
Pacífico/ San Antonio	Male (10)	Not applicable	AdarPC3
Amazonas/ Caño Negro	Female (15)	Not gravid/ not fed	AdarAM1
Amazonas/ Charras	Female (15)	Not gravid/ not fed	AdarAM2
