# Supplementary material for: Metagenomic analysis evidences a core virome in Anopheles darlingi from three contrasting Colombian ecoregions
Source: PLoS One. 2025 Apr 30;20(4):e0320593. doi: 10.1371/journal.pone.0320593 (PMC12043238; doi:10.1371/journal.pone.0320593)
Supplement: S3 Table — (PDF) [file pone.0320593.s004.pdf]

S3 Table. Open reading frames and protein domains in the virus sequences detected in *An. darlingi* natural populations from Colombia.

Virus sequence detected (vOTU)	Pool	ORF length	Protein domains in the sequence (*e-value*) *	RdRp domains (*e-value*) +
Anopheles darlingi rhabdoviridae-like sequence 1	AdarBC1	63aa	6TMR_A Rhabdovirus Glycoprotein (3,5e-4)	N/A
Anopheles darlingi rhabdoviridae-like sequence 2	AdarBC3	103aa	6UEB_A RNA-directed RNA polymerase L (2,9e-27)	Mononeg_RNA _pol (2,7e-28)
Anopheles darlingi rhabdoviridae-like sequence 3	AdarPC3	103aa	Mononeg_RNA_pol super family (2,23e-33)	Mononeg_RNA _pol (1,3e-27)
Anopheles darlingi rhabdoviridae-like sequence 4	AdarBC1	103aa	Mononeg_RNA_pol super family (1,21e-37)	Mononeg_RNA _pol (2,3e-13)
Anopheles darlingi Partitivirus-like sequence 1	AdarBC1, AdarBC2, AdarBC3, AdarBC4, AdarPC1, AdarPC2, AdarPC3, AdarAM1, AdarAM2	265aa	3ES5_B Putative capsid protein (0,33e-7)	N/A
Atrato Partiti-like virus 2 (Capsid)	AdarBC1, AdarBC1	382aa	3ES5_B Putative capsid protein (2,8e-5)	N/A
Atrato Partiti-like virus 2 (RdRp)	AdarBC1, AdarBC2	469aa	RdRP_1 (2,06e-27)	RdRP_1 (2,1e27)
Chibugado virus	AdarBC1, AdarBC3, AdarBC4, AdarPC1, AdarPC2, AdarPC3, AdarAM1	927aa, 423aa, 207aa	RT_LTR (3,47e-76), RNase (3,58e-53), Integrase_H2C2 (7,92e-07), rve (1,78e-06), Gypsy super family (4,08e-23)	RVT_1(2,8e-30)
Atrato Retro-like virus	AdarBC1, AdarPC2, AdarAM1	1375aa	RVT_2 super family (1,03e-72), RNase_HI(1,87e-67), Retrotran_gag_2(2,10e-22)	RVT_2(8,2e-71)
Anopheles darlingi Retro-like virus 1	AdarPC3	1342aa	RVT_2 super family(3,03e66), RNase (4,58e-67), Retrotran_gag_2(7,11e-21)	RVT_2 (2,2e70)
Anopheles darlingi Retro-like virus 2	AdarBC2, AdarBC4	388aa	RNase(3,97e-73), RVT_2 super family (3.16e-32)	RVT_2 (4,3e47)
Anopheles darlingi orbivirus-like sequence	AdarBC1, AdarBC2, AdarBC3, AdarBC4, AdarPC1, AdarPC2, AdarPC3, AdarAM1, AdarAM2	941aa	2BTV_B VP3 CORE PROTEIN (3e-189)	N/A

Anopheles darlingi virus-derived sequence 1	AdarBC1, AdarPC3	164aa	Not detected	N/A
Aedes aegypti To virus 1	AdarBC2, AdarBC3, AdarBC4, AdarPC1, AdarPC2, AdarPC3, AdarAM2	1058aa, 391aa	RT_LTR(1,84e-79), RNase_HI(9,07e-57), Integrase_H2C2, rve	RVT_1(2,9e-06)
Anopheles darlingi To virus 1	AdarBC1, AdarBC2	943aa, 305aa	RT_LTR, RNase(2,72e-59), Integrase_H2C2(2,95e-10), rve(9,53e-04)	RVT_1(1,1e-19)
Anopheles darlingi To virus 2	AdarBC3, AdarBC4, AdarPC1, AdarPC2, AdarPC3, AdarAM1, AdarAM2	932aa, 352aa	RT_LTR(1,51e-80), RNase_HI_RT_Ty3, Integrase_H2C2(2,43e-10), rve(1,23e-05), Gypsy super family(3,88e-68)	RVT_1 (1,5e51)
Anopheles darlingi Chaq virus-like	AdarBC1, AdarBC2, AdarBC3, AdarBC4, AdarPC1, AdarPC2, AdarPC3, AdarAM2	289aa	Not detected	N/A
Murindo virus	AdarPC2	1179aa	Vmethyltransf (9,29e-53), Viral_helicase1(3,64e-42), Peptidase_C21 super family(7,32e-08), psssRNAv_RdRp-like(1,11e-50)	N/D
Anopheles darlingi virus-derived sequence 3	AdarBC3	193aa	5V2S_A Envelope glycoprotein B (6,7e-11)	N/A
Anopheles darlingi orthophasmaviruslike sequence (Nucleocapsid, ORF3) NIRV	AdarAM1, AdarAM2	225aa, 177aa	Not detected	N/A
Anopheles triannulatus orthophasmavirus (Nucleocapsid, ORF3)	AdarAM2	351aa, 106aa	Viral_Nucleoprotein RNA_Complex(17,4e-18)	N/A
Anopheles triannulatus orthophasmavirus (Glycoprotein)	AdarAM1, AdarAM2	403aa	N4HJ1_D ENVELOPE GLYCOPROTEIN(5,1e)-38	N/A
Anopheles triannulatus orthophasmavirus (RdRp)	AdarAM1	1449aa	Bunya_RdRp super family (1,31e-05)	Bunya_RdRp (7,4e-07)

Anopheles darlingi Totiviridae-like sequence	AdarAM1, AdarAM2	228aa	Not detected	N/A

* The conserved domains were identified using the Conserved Domain Database (CDD) and the Protein Data Bank (PDB) with the RPS-BLAST and PHRED tools.

+ The RdRp domains were identified with the HMMER tool.

N/A: not applicable
